# Successful Conservative Management of Uncomplicated Gallbladder Ascariasis

**DOI:** 10.7759/cureus.17160

**Published:** 2021-08-13

**Authors:** Sangharsha Thapa, Kushal Gautam, Swati Chand, Akanchha Khadka, Rasik Neupane

**Affiliations:** 1 Internal Medicine, Kathmandu University School of Medical Sciences, Dhulikhel, NPL; 2 Paediatric Research Unit, Patan Academy of Health Sciences, Kathmandu, NPL; 3 Internal Medicine, Rochester Regional Health, Rochester, USA; 4 Dermatology, MedStar Washington Hospital Center, Washington, USA

**Keywords:** acute abdominal pain, ascaris lumbricoides, ultrasonography, albendazole, biliary ascariasis

## Abstract

Ascariasis is one of the common diseases in human beings worldwide. Most cases are asymptomatic. However, the high parasitic load can present with organ-specific symptoms due to the migration of worms to various regions of the body such as the lungs, nasal cavity, oral cavity, and biliary system, and sometimes with surgical emergencies like intestinal and biliary tree obstruction. Treatment modalities depend on the presentation and the complication associated with it. Uncomplicated cases can be initially managed conservatively and followed up subsequently. However, most cases of biliary ascariasis may require surgical intervention or endoscopic management due to failed management or complications. We report a case of a young male with gallbladder ascariasis diagnosed with ultrasonography and successfully treated with a single dose of albendazole 400 mg. Follow-up ultrasonography was done to evaluate the management.

## Introduction

Ascariasis is a parasitic disease caused by the largest nematode Ascaris lumbricoides (roundworm) [1], which usually infects the small intestine. Its prevalence is high in developing countries with poor hygiene and sanitation. Infection occurs through soil contamination of hands or food, ingestion, and subsequent hatching of eggs in the small intestine [2]. Ascariasis is considered second to gallbladder stones in causing acute biliary symptoms [3]. Most cases are asymptomatic but some might involve the hepatobiliary tree, leading to cholecystitis or cholangitis and a hepatic abscess, considered a medico-surgical emergency [4].

Ultrasound is an essential tool for diagnosing the involvement of the biliary tree but endoscopic retrograde cholangiopancreatography (ERCP) and magnetic resonance cholangiopancreatography (MRCP) are superior with higher diagnostic accuracy [4]. Ultrasonography is preferable because it is easy to handle, cost-effective, non-invasive, and does not use radiation [1]. Treatment modalities depend on the presentation and the complication associated with it. Conservative and surgical management are opted for on the basis of clinical and radiological findings. We present you a case of a young male with ultrasound-confirmed gallbladder ascariasis that was successfully treated with a single dose of albendazole without need for surgery. Follow-up ultrasonography was done to evaluate the progress of the treatment and to rule out cholecystitis and obstruction.

## Case presentation

A 16-year boy presented with epigastric pain for five days, which was sudden in onset, colicky, and lasting for five to 10 minutes. There was no radiation, and no aggravating and relieving factors. He also gave a history of two episodes of non-bilious vomiting. There was no history of abdominal distension, water brash, nausea, chest pain, fever, and headache. Bladder and bowel habit was normal. His past history was insignificant. General examination was normal and vitals were within the normal range. On abdominal examination, right upper quadrant with guarding, tympanic note on percussion, and normal bowel sounds on auscultation were noted. Other systemic examinations were normal. Routine blood investigation showed total leukocyte count 15000/ul (neutrophils - 70%, lymphocytes - 27%, monocytes - 2%, eosinophils - 1%), hemoglobin 11 gm/dl, packed cell volume 31%, and platelets 268000/ul. Liver function tests showed total bilirubin 0.6 mg/dl, direct bilirubin 0.1 mg/dl, aspartate aminotransferase 512 IU/L, alanine aminotransferase 261 IU/L, and alkaline phosphatase 217 IU/L. Urine, stool routine, and microscopic examination were unremarkable, and no larvae or ova of intestinal parasites were found.

**Figure 1:**
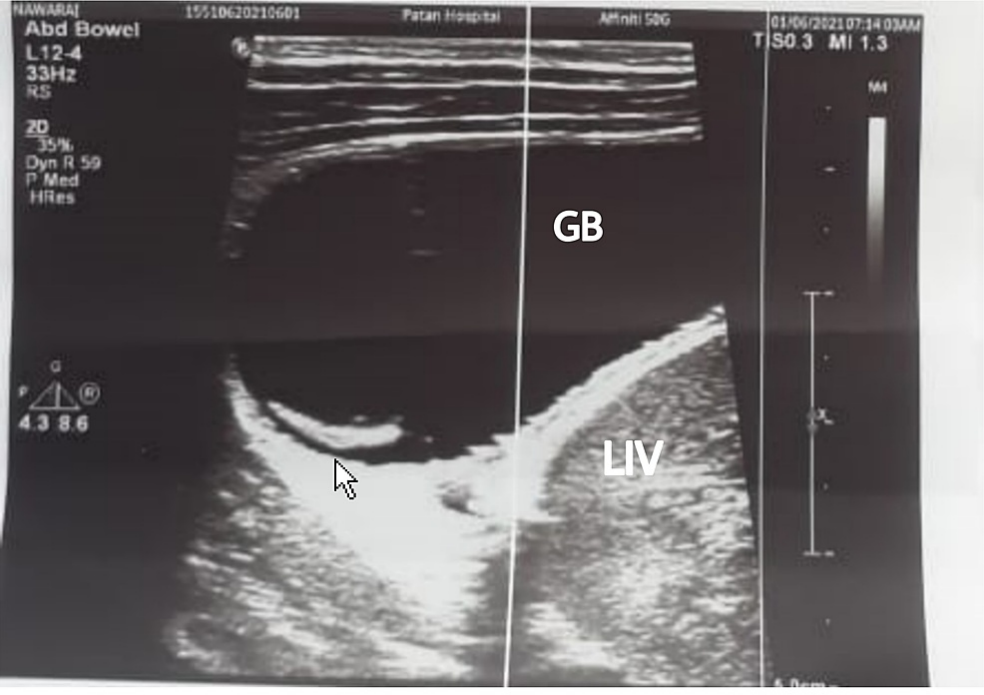
A 16-year-old male with acute abdominal pain showing a worm inside the gall bladder Note the distended gallbladder with linear echogenic mobile structure within the lumen (white arrowhead).

Ultrasonography of abdomen showed distended gallbladder with linear echogenic mobile structure within the lumen, suggestive of worm infestation as shown in Figure 1.

**Figure 2:**
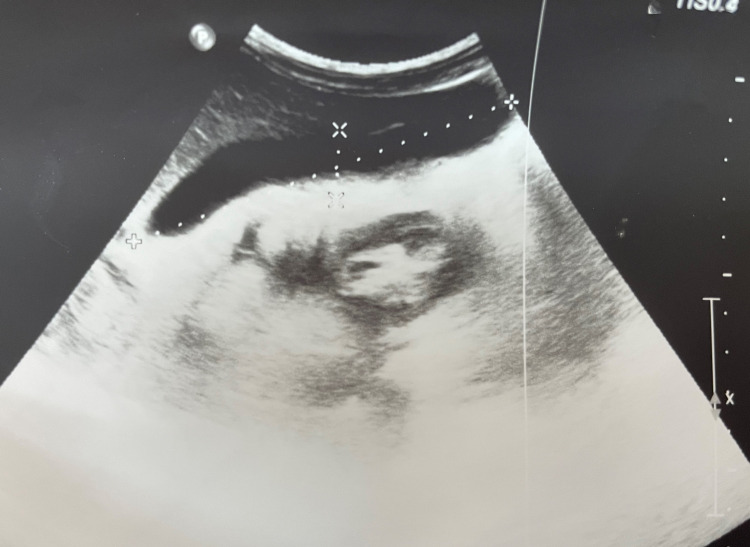
Normal ultrasonography of gallbladder suggesting removal of the worm from the gallbladder

He was medically managed with IV fluids, injection pantoprazole, ondansetron, and deworming with albendazole 400 mg. His condition gradually improved and epigastric pain gradually subsided. Abdominal ultrasonography five days after the deworming did not reveal any worm or inflammatory changes in the gall bladder and common bile duct (CBD) shown in Figure 2. The patient showed complete recovery and was discharged. Follow-up after a week did not reveal any signs and symptoms suggestive of obstruction and cholecystitis.

## Discussion

Ascaris lumbricoides, the largest nematode (roundworm) that usually infects the small intestine is considered one of the most common parasitic infections in the world, mostly in developing countries with poor hygiene and sanitation. Around 1.4 billion people have been infected with ascariasis and most of them belong to developing countries, which have moist soil [5]. About 70% of children are found to be infected in tropical areas [5]. In most endemic areas like developing countries and tropical areas, it is most likely transmitted from person to person [6]. Infection occurs through soil contamination of hands or food, ingestion, and subsequent hatching of eggs in the small intestine. Usually, it takes five to 10 days for the fertilized eggs of a worm to become infectious [7]. Eggs may remain viable for 17 months.

Most ascariasis-infected patients remain asymptomatic. However, it has a significant effect with different modes of presentation in the hepatobiliary system. Simply, worm infestation can present with biliary colic due to the entry of the worm into the biliary tree from the duodenum through the ampullary orifice. In some cases, it may present with acute cholecystitis or cholangitis, considering as a medico-surgical emergency [8-9]. Dead ova released from the female worm may lead to a granulomatous inflammatory reaction causing the hepatic abscess [8]. It is commonly seen in children. In an Indian study from Kashmir, at the highly endemic area, ascariasis was found to be the cause in 36.7% of cases of 109 patients with proven biliary and pancreatic disorders [10].

Diagnosis depends on the clinical settings where physicians are practicing. In most of the tropical areas and endemic areas likely developing countries have less advanced imaging modalities to diagnose the worm manifestation in the biliary tract. Therefore, radiological imaging, such as USG, is becoming increasingly common and promoted as an initial imaging modality in the diagnosis of biliary ascariasis [9,11].

For biliary ascariasis, the recommended imaging is USG, MRCP, or ERCP [9,11]. Ascariases in the biliary system are seen as long, linear, or curved echogenic structures without acoustic shadowing with writhing movements appreciated in real-time ultrasonography [8]. Compared with ERCP, ultrasonography has high sensitivity in diagnosing ascarids in the bile ducts [8]. Better imaging modalities, such as MRCP, endoscopic ultrasound, or ERCP are indicated in cases with high clinical suspicion [9-11]. In our case, we avoided invasive procedures as we were able to diagnose the worm on the basis of ultrasonographic findings.

Treatment modalities depend on the presentation and the complication associated with it. Conservative and surgical management have opted on the basis of clinical and radiological findings. Conservative management can be tried initially under close watch in uncomplicated cases or when surgical management is not feasible or denied. Endoscopic or surgical worm removal should be done when there is the presence of worms trapped in the biliary tree or invasion of the liver by one or more worms and if the patient doesn't respond to conservative management [4,12]. We managed our case conservatively, as there was no associated complication with biliary ascariasis. Although a number of antihelminthic drugs, such as pyrantel pamoate, mebendazole, ivermectin, and levamisole, have been used to effectively manage ascariasis [8], a single dose of albendazole or mebendazole is usually considered as the effective agent [13]. In our case, anti-helminth (albendazole) was tried, and the patient was treated effectively without any complications. Follow-up USG and evaluation were done to ensure the adequacy of the treatment.

## Conclusions

Biliary ascariasis is a complication of intestinal ascariasis that can cause life-threatening manifestations. A high index of suspicion in the endemic areas along with ultrasonography is the key to diagnosis. Medical management can be tried in uncomplicated cases. However, close observation for the adequacy of the treatment and possible complications must be done in subsequent follow-ups. Deworming can treat asymptomatic intestinal cases and prevent surgical emergencies in endemic regions. Meanwhile, improvement in sanitation plays a crucial role in the epidemiological control of hepatobiliary disease.
